# Gastric Polyposis Due to Foreign Bodies and *H. pylori* Infection: Case Report and Literature Review

**DOI:** 10.3390/reports9010084

**Published:** 2026-03-12

**Authors:** Cătălina Dănilă, Lucian Mocan, Ovidiu Laurean Pop, Andrea Pop-Crisan, Lucian Faur, Simona Daniela Cavalu

**Affiliations:** 1 Faculty of Medicine and Pharmacy, University of Oradea, P-ta 1 Decembrie 10, 410073 Oradea, Romania; danila.catalina@student.uoradea.ro (C.D.); drovipop@gmail.com (O.L.P.); andrea20_crisan@yahoo.com (A.P.-C.); ld_faur@yahoo.com (L.F.); 2 3rd Surgery Clinic, “Iuliu Hatieganu” University of Medicine and Pharmacy, 400012 Cluj Napoca, Romania; lucian.mocan@umfcluj.ro

**Keywords:** endoscopy, foreign body ingestion, gastric polyps, *H. pylori* infection

## Abstract

**Background and Clinical Significance:** Foreign body ingestion represents an endoscopic emergency, with a risk of organ perforation of up to 35%, where increased prevalence was noticed among people with mental disorders and institutionalized patients. **Case Presentation**: The patient—male, 23 years old, and institutionalized for sequelae of infantile encephalopathy—was admitted for epigastric pain and hyperemetic syndrome that began 10 days earlier. Endoscopically, 12 hard plastic foreign bodies with sharp edges and sizes of 6–7 cm were identified, followed by extraction that was successfully performed in two sessions using a polypectomy snare and a Foreign Body Hood Protector. Additionally, multiple sessile exulcerated polypoid lesions were observed, measuring around 1–3 cm each, occupying the entire antrum. Histological examination showed inflammatory/regenerative elements, with features of moderate-to-high-grade dysplasia, while a rapid urease test for *Helicobacter pylori* infection was positive. As a consequence, the patient was administered triple eradication therapy. In addition, the patient presented marked features of hypereosinophilia and splenomegaly. Upon endoscopic reevaluation after 3 years and 8 months, no polyps were present and the *H. pylori* test was negative, while a complete and spectacular remission of both the hypereosinophilia and splenomegaly was observed. **Conclusions:** This case illustrates that the development and progression of gastric polyposis may be caused by the coexistence of chronic mucosal irritation from foreign bodies and *H. pylori* infection, which is a rare association. *H. pylori* eradication and endoscopic removal of the foreign bodies resulted in significant mucosal improvement.

## 1. Introduction and Clinical Significance

Gastric polyps are a diverse group of lesions arising from the gastric mucosa or submucosa, encompassing hyperplastic, inflammatory, fundic gland, adenomatous, and serosal/foreign-body-associated variants [1]. Foreign bodies introduced into the stomach—whether iatrogenic, accidental, or intentional—can produce a spectrum of endoscopic appearances. Chronic irritation from a retained object may lead to reactive granulation tissue, inflammatory polyps, or focal mucosal hyperplasia. In some cases, the foreign material may be encapsulated or partially embedded within a polyp, necessitating careful histopathologic evaluation to differentiate benign reactive changes from dysplasia or neoplasia. The clinical significance lies not only in an accurate diagnosis but also in determining whether removal of the foreign body is indicated and how mucosal healing impacts polyp dynamics [2].

Clinically, patients may present with nonspecific epigastric discomfort, early satiety, anemia, or incidental findings on imaging or endoscopy performed for unrelated reasons. Endoscopic assessment is essential for characterizing the lesions, assessing the relationship to the foreign body, and determining the feasibility of endoscopic retrieval or need for surgical intervention. An endoscopic biopsy can help distinguish reactive polyps from neoplastic processes. The disadvantage consists in the likelyhood of limited access to biopsy with precision, especially if the lesion is fibrous or adherent to the foreign material. In select cases, imaging and endoscopic ultrasound may aid in assessing the depth of involvement and surrounding tissue reaction [3].

In the context of a gastric foreign body, the management focuses on two parallel goals: (1) safe and definitive removal or stabilization of the foreign object to prevent ongoing injury, migration, or perforation, and (2) appropriate treatment of the resulting mucosal lesion. If the foreign body is removable endoscopically and the polyp is purely reactive without dysplasia, removal plus a targeted biopsy may suffice, followed by surveillance to document mucosal recovery. If the polyp demonstrates dysplasia or neoplastic features, or if the foreign body cannot be safely retrieved endoscopically, multidisciplinary discussion regarding surgical options is warranted. Histopathology remains crucial to distinguish inflammatory or reparative polyps from neoplastic processes and to assess for complications such as ulceration or metaplasia [4].

Hypereosinophilia, defined as an elevated peripheral eosinophil count that is persistently above the expected reference range, reflects a spectrum of disorders ranging from allergic and parasitic processes to primary hematologic diseases and eosinophilic GI involvement. While eosinophilia is commonly discussed in the context of asthma, dermatologic conditions, or hypereosinophilic syndromes, its intersection with gastric foreign bodies is a less familiar but clinically meaningful topic [5]. This case-based discussion explores the potential link between gastric foreign bodies and eosinophilic gastric mucosal responses, highlighting diagnostic considerations, pathophysiologic mechanisms, and therapeutic implications.

Gastric foreign bodies can provoke a localized inflammatory environment characterized by mucosal injury, chronic irritation, and regenerative changes. In susceptible individuals, this environment may recruit eosinophils to the gastric mucosa, contributing to endoscopic findings such as edema, friability, and polypoid changes, as well as histologic features including eosinophilic infiltration of the lamina propria and muscularis mucosae [6]. Eosinophil-dominated responses in the stomach may be driven by several pathways: direct tissue injury from the foreign body, mechanical stimulation, microbiome-epithelial interactions, and systemic or regional allergic or hypereosinophilic processes. Distinguishing a reactive, localized eosinophilic response from systemic eosinophilic gastroenteritis or other eosinophil-driven diseases is essential, as it guides both the management of the foreign body and any targeted anti-eosinophilic therapies [7].

The present case highlights educational themes applicable to gastroenterology practice: recognizing atypical etiologies of gastric polyps, integrating endoscopic and histopathologic data in the setting of a foreign body, and balancing the risks and benefits of foreign-body removal with lesion-directed therapy. It also illustrates decision-making frameworks for selecting surveillance intervals, determining indications for retrieval, and anticipating mucosal recovery after foreign-body removal.

The patient’s history of foreign-body ingestion, endoscopic findings, histopathologic results, imaging studies, and the therapeutic approach chosen provided the outline for the present case. We discuss the rationale for foreign-body management in conjunction with polyp treatment and review relevant guidelines on endoscopic removal and surveillance of reactive gastric lesions, reflecting on implications for prognosis and follow-up. Additionally, through a literature review, we aim to illuminate the pathophysiology of foreign-body-associated gastric polyps and provide a practical framework for evaluation and management of similar scenarios in clinical practice.

## 2. Case Presentation

### 2.1. General Data

This is the case of a male, 23 years old, who was admitted three times in our service (initial evaluation and two follow-ups), being diagnosed with infantile encephalopathy and intellectual disability, and also presenting trouble concerning his communication skills.

### 2.2. Clinical Data

Upon the first admission, the patient presented nausea and abdominal pain with 10 days prior onset. He was normal weight and had a slim abdomen, which presented tenderness upon palpation in the epigastric region. Mainly because of the communication limitations, multiple laboratory tests, imaging tests and endoscopies were performed on each admission.

### 2.3. Endoscopy Results

**Figure 1:**
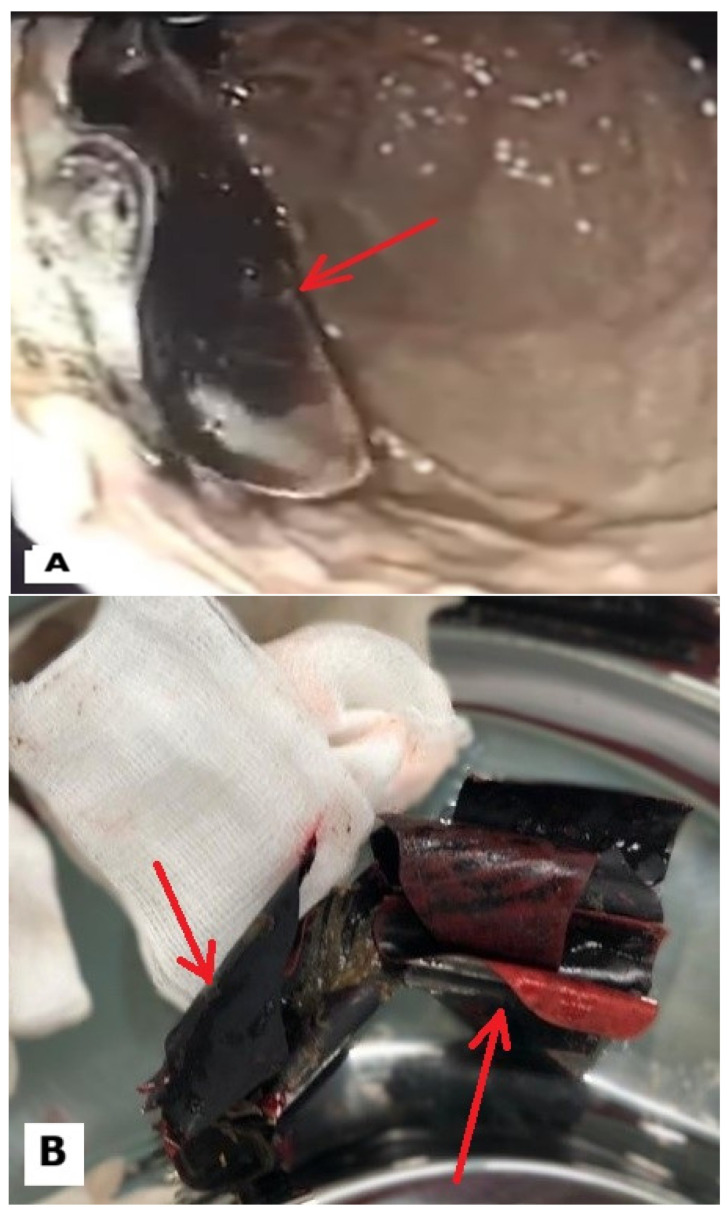
(**A**) Sharp gastric foreign object observed during the first endoscopy; (**B**) two sharp foreign objects after endoscopic retrieval.

A total of three upper digestive tract endoscopies were performed (one during the initial admission and the others during the follow-ups). The endoscopy team was surprised to discover around 12 foreign plastic objects that were around 7–8 cm in length; most were blunt, but some had sharp edges, as depicted in Figure 1A,B. There was no data regarding when they were swallowed or whether they were swallowed at the same time.

**Figure 2:**
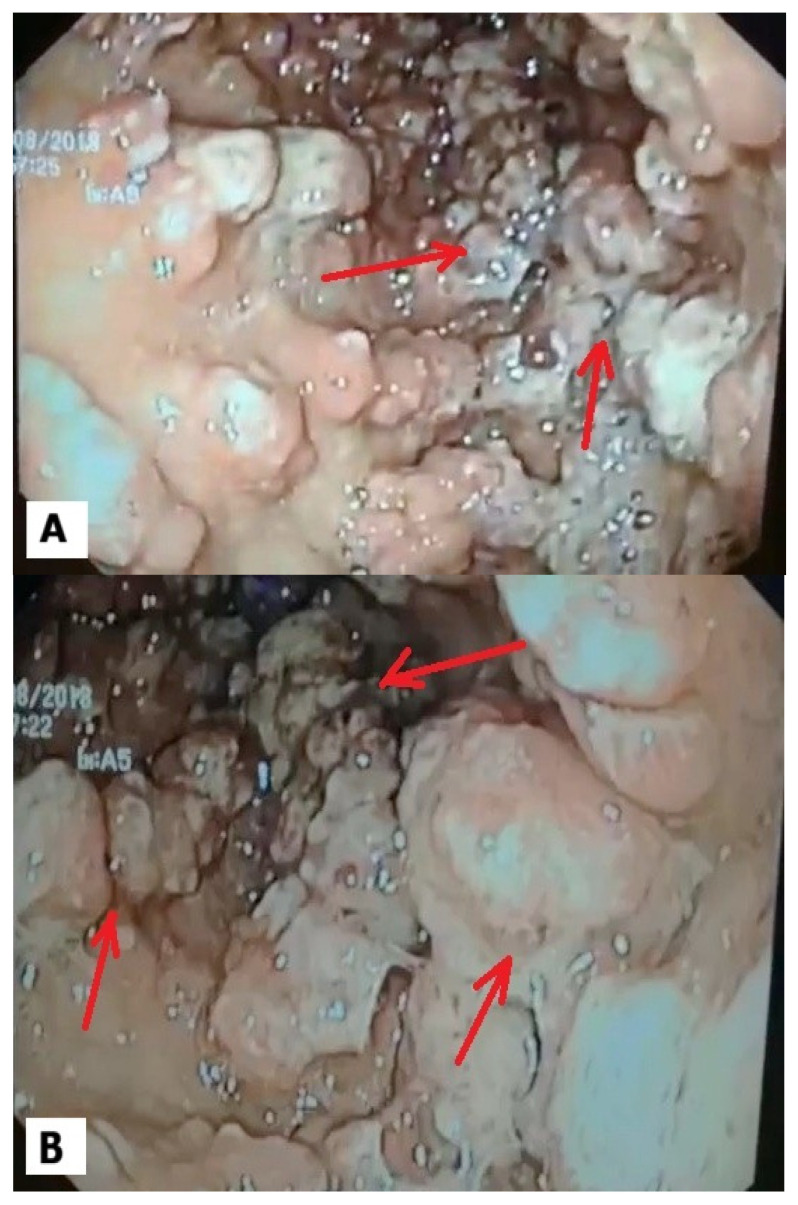
(**A**) Gastric body with ulcerated sessile polyps; (**B**) antrum with ulcerated sessile polyps.

The whole gastric body and antrum was covered with polypoid sessile lesions with an adenomatous appearance, measuring 1–3 cm, as illustrated in Figure 2A,B. Their texture was firm when biopsies were performed. Because of the risk of organ perforation, the sharp objects were immediately extracted. The blunt objects that did not present a risk of organ perforation or occlusion were extracted during the follow-up endoscopy. A rapid urease test was performed and showed the presence of an *H. pylori* infection, and hence, a regular regimen of eradication therapy was initiated and consisted of PPI, amoxicillin and levofloxacin [8].

**Figure 3:**
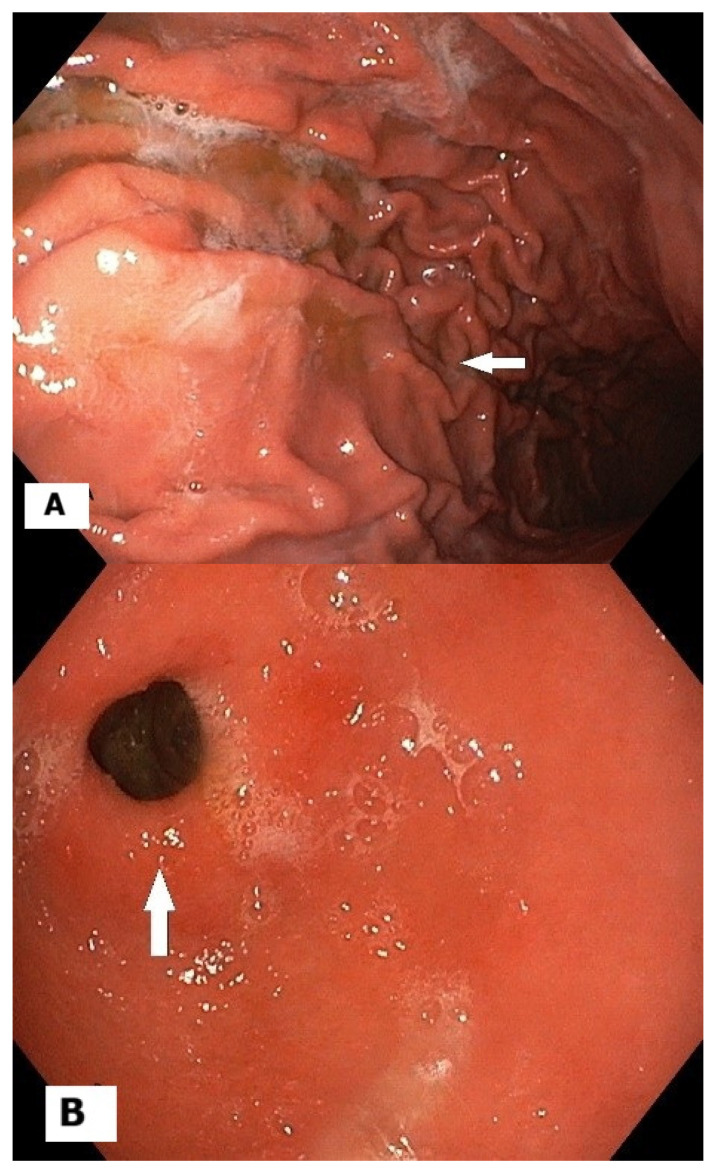
(**A**) Gastric body folds at final follow-up; (**B**) gastric antrum mucosa at final follow-up.

The third and last follow-up endoscopy revealed chronic gastritis without any polyps, as seen in Figure 3, while the rapid urease test was negative.

### 2.4. CT Scan

**Figure 4:**
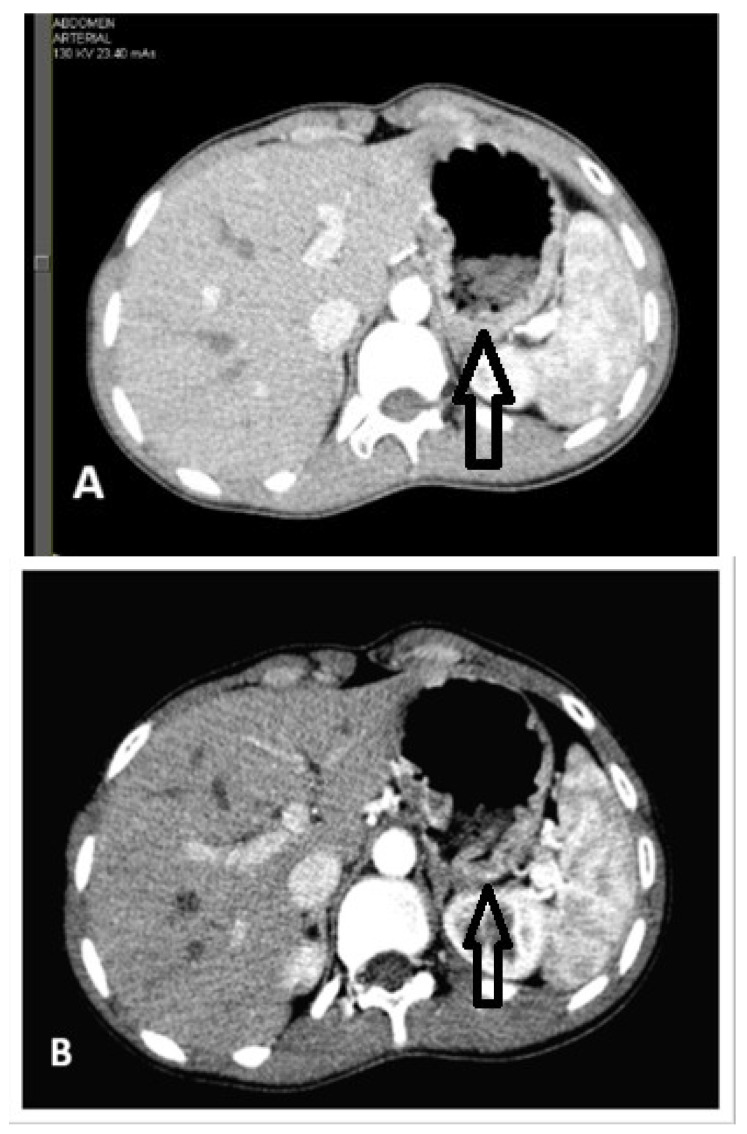
(**A**) Abdominal CT examination, arterial phase (arrow depicting moderate contrast intake and gastric wall thickening). (**B**) Abdominal CT examination, venous portal phase (arrow depicting gastric wall thickening).

Given the presence of multiple large-sized gastric polyps, a CT scan was performed, showing irregular thickening of the gastric walls with moderate contrast uptake and mild splenomegaly and without perigastric adenopathies (Figure 4).

### 2.5. Histopathology Results

**Figure 5:**
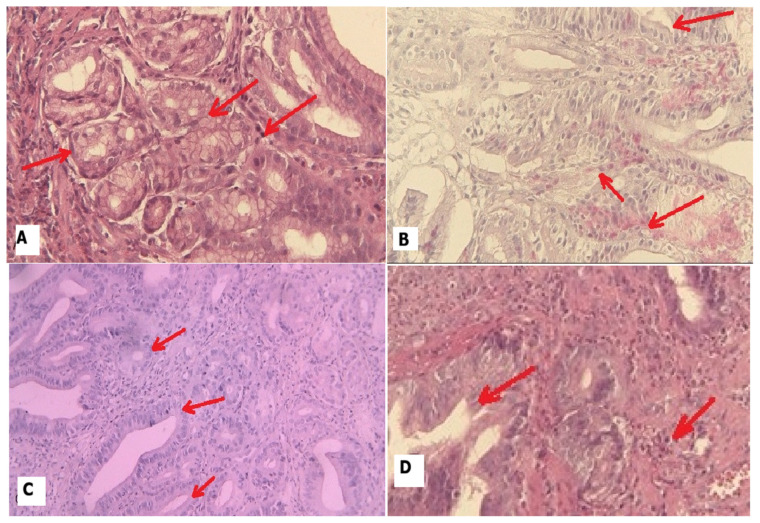
(**A**) The section shows gastric mucosa with complex glandular crowding and slight architectural distortion. The epithelium demonstrates nuclear enlargement, hyperchromasia, and focal loss of polarity with increased nuclear-to-cytoplasmic ratio. No unequivocal invasion beyond the basement membrane is visible (20× objective). (**B**) This panel highlights gastric mucosa with mixed inflammatory infiltrate, including lymphocytes, plasma cells, and focal eosinophils. The epithelium shows dysplasia with nuclear stratification and surface maturation loss. The surrounding lamina propria is expanded by chronic active inflammation, consistent with ongoing mucosal injury (20× objective). (**C**) Gastric mucosa continues to show architectural abnormalities, with crowded, elongated, and irregularly branching glands. Cytologic atypia remains evident, including nuclear pleomorphism and chromatin clumping. The inflammatory infiltrate persists but appears slightly reduced compared with earlier sections. No features of invasion are identified (20× objective). (**D**) The mucosa now displays preserved glandular architecture, normal surface maturation, and the absence of cytologic atypia. Only minimal chronic inflammation persists, with no dysplastic features. There is no erosion, ulceration, or architectural complexity. The overall histology is consistent with the complete regression of previously dysplastic lesions (20× objective).

During the first and second endoscopies, multiple biopsies were collected from the polyps and sent for histopathological examination. Hematoxylin–eosin staining was performed, showing both inflammatory and mild dysplasia lesions, as illustrated in Figure 5A–C. During the last follow-up endoscopy, a final set of biopsies were collected and the histopathologist concluded that only inflammatory lesions were present, as illustrated in Figure 5D. Giemsa staining was also performed and showed that *H. pylori* was no longer present [9].

### 2.6. Laboratory Results and Other Investigations

Baseline laboratory investigations indicated marked eosinophilia, where this abnormality was sustained and reached its zenith during the second assessment. The hematological findings were assessed in conjunction with the observed mild splenomegaly, and consequently, a hematologist performed a bone marrow biopsy, which was negative for malignancy.

Table 1 presents the evolution of the eosinophile count from the moment of diagnosis to the last follow-up. The spike point was recorded on September 2018, marking the final endoscopic extraction of the foreign bodies. The endoscopies were performed on April 2018, September 2018 and January 2022, each one on the same day as the admission. Subsequently, the eosinophile count began to decrease, reaching normal values by January 2025.

## 3. Discussion

This case describes a unique constellation of gastric pathology following ingestion of multiple foreign bodies, concurrent *Helicobacter pylori* infection, and marked hypereosinophilia. The clinical intervention consisted of removal of intragastric foreign bodies with subsequent eradication of *H. pylori*, leading to full remission despite the high dysplasia. This context offers several points of discussion regarding pathogenesis, diagnostic strategy, management, and prognostic implications.

The attempt to establish the determining cause of the gastric polyps from a morphological and histopathological perspective is difficult. They could be primarily caused by chronic irritation from the foreign bodies or by *H. pylori* infection. Histopathology results in this case can find characteristics that occur both in chronic *H. pylori* infection and persistent mucosal injury. These findings may include foveolar hyperplasia, glandular distorsion or inflammatory cell infiltration.

The idea that the *H. pylori* infection served as the main cause of mucosal proliferation and possibly produced a synergistic inflammatory impact in addition to the mechanical irritation from the foreign bodies is strongly supported by the significant regression of the patient’s polypoid lesions.

Table 2 summarizes literature case reports regarding the relationship between foreign bodies and gastric polyps and lesions, along with the main features and outcomes.

The development of multiple gastric polyps secondary to chronic foreign body ingestion is an exceptionally rare phenomenon, with limited reports in the literature.

Our patient presented with multiple polypoid lesions visualized using endoscopy, which resolved completely following the removal of the foreign bodies and appropriate medication. These findings suggest a sinergic causal relationship between the chronic presence of foreign objects in the stomach, *Helicobacter pylori* infection and polyps formation.

The literature on foreign-body-related gastric polyps is largely limited to case reports describing polypoid lesions or mass-like granulomas resulting from chronic mucosal irritation. Daneshbod et al. (2011) reported a case of a peach kernel lodged in the duodenum that mimicked a polypoid tumor endoscopically and was initially misdiagnosed as malignancy before histological analysis revealed no neoplasia but foreign-body-induced inflammation [10]. Okasha et al. (2024) also documented a fishbone-induced gastric granuloma masquerading as malignancy [11]. These cases highlight the potential for foreign bodies to induce chronic inflammatory reactions that mimic polypoid lesions or tumors, though they do not represent true epithelial polyps.

In contrast, the coexistence of a true gastric polyp in association with a foreign body is rare and not well established as causative. Behzad et al. (2020) reported a case of a gastric inflammatory fibroid polyp alongside a foreign-body-induced liver abscess, concluding that the occurrence was coincidental rather than causative [12].

The mechanism by which foreign bodies may induce polypoid lesions likely involves chronic mechanical irritation leading to localized mucosal inflammation, hyperplasia, and regenerative changes. These inflammatory changes can give rise to polypoid appearances, granulomas, or pseudotumoral lesions on endoscopy. Our case presented unique features, with multiple polyps that resolved entirely following the foreign body removal and medical treatment, suggesting these lesions were reactive and reversible rather than true neoplastic polyps.

On the other hand, *Helicobacter pylori* infection has been implicated in the pathogenesis and persistence of gastric hyperplastic polyps, with multiple studies reporting regression of polyps following eradication therapy [16,17]. The current literature suggests that *H. pylori* induce a hyperproliferative state via inflammatory cytokines; thus, the mechanism of polyp remission following eradication therapy is likely attributable to the cessation of this inflammatory cascade [18]. Ohkusa et al. demonstrated the disappearance of hyperplastic polyps after successful eradication [19], suggesting a causal role for infection. Subsequent observational work consistently linked *H. pylori* eradication with polyp regression [20,21]. Several studies pointed out that eradication reduces the recurrence risk after endoscopic resection [22,23], reporting lower recurrence rates post-eradication; drug therapy targeting *H. pylori* in infected polyps also favorably affected outcomes [24]. Collectively, these data support incorporating *H. pylori* testing and eradication as part of the management strategy for patients with gastric hyperplastic polyps, especially in the context of endoscopic resection, with a follow-up endoscopy to document regression and monitor recurrence. Table 3 summarize the most relevant publications regarding gastric polyps regression after *H. pylori* eradication.

Data on the regressions of low- and high-grade dysplasias after eradication of *H. pylori* infection are very scarce.

The current literature presents meta-analyses on the regression of precancerous lesions (such as atrophic gastritis, along with focal or complete type of intestinal metaplasia) and gastric polyps with eradication of *Helicobacter pylori* infection, but there is no evidence of moderate dysplasia without resection of lesions [28,29,30]. Piazuelo and collaborators demonstrated that after at least six years without infection, a preneoplastic lesion such multifocal atrophic gastritis is reversible to a non-atrophic state, while the protective effect of anti-*H. pylori* therapy (ITT) against progression of precancerous lesions remains after 20 years. In the initial years of follow-up, bacterial clearance did not result in any discernible histological changes, according to analysis of the cumulative effect of *H. pylori* status from baseline to 20 years. However, as the number of years without infection increased, more significant consequences were observed [31].

Several authors reported that eradication of *Helicobacter pylori* infection decreases the risk of metachronous gastric cancer after removal of dysplastic lesions [32,33].

Two cases of low and moderate gastric epithelial dysplasia with *H. pylori* infection have been reported in the literature. Following *H. pylori* eradication, there is evidence of both significant endoscopic expression and regression of histological lesions, after one, two and three years follow-up, while gastric dysplasia was observed at the 3-month follow-up [34].

Given the unique features of the present case, our findings underscore the importance of considering foreign body ingestion in the differential diagnosis of multiple gastric polyps, particularly when the clinical history is suggestive and when other common etiologies are excluded. Endoscopic surveillance following foreign body extraction is recommended to confirm resolution of mucosal abnormalities.

Future studies comprising large number of cases are necessary to be documented to further characterize the pathophysiological mechanisms linking chronic foreign body presence to gastric mucosal proliferation and polyp formation. Until then, clinicians should maintain a high index of suspicion for foreign-body-induced mucosal lesions in atypical cases.

Ultimately, our report provides proof that eliminating the mechanical irritant and the antigenic stimulation, *H. pylori* can result in the full restoration of the stomach architecture, providing young patients with complicated comorbidities with an organ-sparing prognosis.

## 4. Conclusions

The development of inflammatory-driven dysplastic gastric lesions is an example of the synergistic pathogenic interaction between persistent *Helicobacter pylori* infection and prolonged mechanical irritation from retained foreign materials. A case of *H. pylori* infection and diffuse gastric lesions has been illustrated in this report as a rare association. The gastric lesions significantly regressed after the endoscopic removal of foreign bodies combined with *H. pylori* eradication. This case suggests that gastric mucosal pathology may be aggravated by the simultaneous presence of chronic mechanical irritation and infection.

## Figures and Tables

**Table 1:** Comparison of blood count and endoscopy results.

Test	Normal Range	April 2018	June 2018	September 2018	January 2022	Janurary 2025
White blood cells	4.00–10.40 × 10^3^/µL	10.9	12.6	5.5	6.25	5.04
Red blood cells	4.3–15.7 × 100^3^/µL	3.5	4.38	4.01	4.10	4.46
Hemoglobin	13.0–17.0 g/dL	8.6	12.8	12.2	11.9	12.7
Hematocrit	40–52%	26.8	37.3	35.6	36.3	38.2
MCV	80–96 fL	76.7	85.1	88.8	88.4	85.6
MCH	26–32 pg	24.7	29.1	30.4	29.1	28.5
MCHC	32–36 g/dL	32.2	34.2	34.2	32.9	33.2
Platelets	150–400 × 100^3^/µL	595	322	243	357.1	302.1
Neutrophiles%	45–72%	42.3%	16.7%	35.7%	66.2%	62.8%
Lymphocites%	25–38%	17.5%	17.4%	37.9%	22.6%	24.5%
Monocites%	0–8%	4.8%	3.1%	7.6%	7.1%	10.0%
Eosinophiles%	0–5%	34.8%	62.5%	18.4%	3.1%	2.4%
Basofiles%	0–1%	0.6%	0.3%	0.4%	0.2%	0.21%
Neutrophiles	1.8–7.5 × 10^3^/µL	4.6	2.1	2.0	4.1	3.16
Lymphocites	1–4 × 10^3^/µL	1.9	2.2	2.1	1.4	1.24
Monocytes	0–0.8 × 10^3^/µL	0.5	0.4	0.4	0.4	0.505
Eosinophiles	0–0.5 × 10^3^/µL	3.8	7.9	1.0	0.1	0.121
Basofiles	0–0.1 × 10^3^/µL	0.1	0.0	0.0	0.0	0.011
Gastroscopy		Gastric polyposis and erosions	-	Gastric polyposis and erosions	Antral erithema	-
Histopathology		Low-grade/moderate-grade dyslpasia	-	Low-grade/moderate-grade dysplasia	*H. pylori* negative gastritis	-

**Table 2:** Literature review of the most relevant aspects of gastric lesions due to foreign bodies.

Nr.	First Author/Year/Ref.	Article Type	Main Features and Case Particularities	Diagnostic Tools and Clinical Interventions	Main Outcomes
1.	Daneshbod et al., 2011 [10]	Case report	A duodenal foreign body (peach kernel) mimicked a gastric polypoid tumor on endoscopy. Biopsy misread as malignancy.	Endoscopy, biopsy, and surgery	Shows how FBs can mimic gastric polyps; important diagnostic consideration. No true polyp formation observed.
2.	Okasha et al., 2024 [11]	Case report	Gastric granuloma formed around a fishbone; lesion appeared polypoid/submucosal on EUS.	Endoscopic ultrasound and histopathology	Demonstrates FB-induced granulomatous reaction resembling polyp. Not a true epithelial polyp.
3.	Behzad et al., 2020 [12]	Case report	Coexistence of a gastric inflammatory fibroid polyp (IFP) and a liver abscess caused by FB. No direct causal link shown.	Endoscopy and histopathology	Suggests possible inflammatory polyp in setting of FB-related inflammation, but authors concluded coincidence.
4.	Shen et al., 2025 [13]	Case report	Hair ingestion caused a gastric perforation with FB granuloma formation. Appeared as submucosal mass.	Imaging, surgery, and pathology	Granulomatous tissue mimicked mass lesion; relevant as differential for FB-associated pseudopolyps.
5.	Rossi et al., 2012 [14]	Case report and literature review	Inflammatory fibroid polyps described in stomach. No link to FBs, but important differential diagnosis for submucosal gastric lesions.	Review of histologic cases	Establishes typical IFP appearance vs. FB-related granulomas; useful for comparison.
6.	David H Dupont et al., 2024 [15]	Case report	Fish bone impactation of stomach and colon, as well as microperforation of the small bowel.	CT scan, gastroscopy, and colonoscopy	Non-surgical management even if organ perforation was present.

**Table 3:** Role of *H. pylori* eradication in gastric polyps regression.

Nr.	Author(s), Ref.	Year	Study Design	Key Findings	Methodology	Relevance
1	Ohkusa T, Fujiki K, Takashimizu I, et al. [19]	1998	Prospective cohort	Most hyperplastic polyps disappeared after *H. pylori* eradication	Observational follow-up of patients with hyperplastic polyps before and after eradication	Shows regression of polyps after eradication; supports eradication as first-line intervention
2	Nam SY, Park BJ, Ryu KH, et al. [25]	2016	Prospective cohort	Significantly higher regression rates of hyperplastic polyps in patients with eradication vs. persistent infection	Follow-up endoscopy of *H. pylori*-positive patients with hyperplastic polyps	Confirms eradication efficacy in Korean population
3	Ouyang Y, Li Y, Ma Y, et al. [20]	2021	Meta-analysis	*H. pylori* eradication markedly increases odds of complete regression of hyperplastic polyps	Systematic review and meta-analysis of published trials	High-level evidence for eradication effect; provides quantitative support
4	Elhanafi S, Saadi M, Lou W, et al. [21]	2015	Cross-sectional	Hyperplastic polyps associated with *H. pylori*; fundic gland polyps inversely associated	Endoscopic biopsy and histopathology analysis	Highlights polyp type-specific associations with *H. pylori*; relevant for differential diagnosis
5	Cho Y-S, Chung W-C, Kim J-Y, et al. [22]	2022	Cohort post-EMR	Recurrence of hyperplastic polyps significantly lower after eradication	Endoscopic mucosal resection followed by repeated endoscopy	Supports eradication to prevent recurrence post-polypectomy
6	Kang KH, Hwang SH, Kim D, et al. [23]	2018	Retrospective cohort study	Recurrence higher with persistent *H. pylori* (42.9%) vs. eradicated/negative (18.9%)	Follow-up after EMR; *H. pylori* status assessed	Shows importance of eradication in preventing recurrence after polyp removal
7	Nam SY, Park BJ, Ryu KH, et al. [26]	2020	Prospective cohort	Disappearance rate: 83.7% after eradication vs. 34.1% non-eradicated	Screening cohort with follow-up endoscopy of polyps <10 mm	Strong evidence of eradication effect in population screening context
8	Nam SY, et al. [27]	2020	Randomized controlled trial	All patients in eradication arm showed regression; none in control arm	Open-label single-center RCT; follow-up endoscopy	Confirms causality between eradication and polyp regression
9	Woo Chul Chung [22]	2023	Prospective observational study	*H. pylori* eradication reduces recurrence of gastric hyperplastic polyps after EMR; recurrence: 8.1% (eradication) vs. 19.2% (non-eradication)	18-month follow-up; comparison between eradication and non-eradication groups	Supports *H. pylori* eradication to prevent recurrence in gastric hyperplastic polyps post-EMR
10	Ji F, Wang Z-W, Ning J-W, et al. [24]	2006	Randomized controlled trial	Treatment group: 68% polyp disappearance at ~6.5 months; controls had no change	PPI + clarithromycin + bismuth + tinidazole; follow-up endoscopy	Provides controlled evidence of pharmacologic eradication efficacy for small hyperplastic polyps

## Data Availability

All data are available in the County Emergency Clinical Hospital of Oradea—Bihor, Romania, including databases and consultation registers from the Gastroenterology Department, and the paraffin-embedded tissue blocks from the Pathology Department.
